# Vector-free intracellular delivery by reversible permeabilization

**DOI:** 10.1371/journal.pone.0174779

**Published:** 2017-03-30

**Authors:** Shirley O’Dea, Valeria Annibaldi, Louise Gallagher, Joanne Mulholland, Emer L. Molloy, Conor J. Breen, Jennifer L. Gilbert, Darren S. Martin, Michael Maguire, Fitz-Roy Curry

**Affiliations:** 1 Avectas Ltd., Maynooth, Co. Kildare, Ireland; 2 Department of Physiology & Membrane Biology, University of California, Davis, California, United States of America; Laurentian, CANADA

## Abstract

Despite advances in intracellular delivery technologies, efficient methods are still required that are vector-free, can address a wide range of cargo types and can be applied to cells that are difficult to transfect whilst maintaining cell viability. We have developed a novel vector-free method that uses reversible permeabilization to achieve rapid intracellular delivery of cargos with varying composition, properties and size. A permeabilizing delivery solution was developed that contains a low level of ethanol as the permeabilizing agent. Reversal of cell permeabilization is achieved by temporally and volumetrically controlling the contact of the target cells with this solution. Cells are seeded in conventional multi-well plates. Following removal of the supernatant, the cargo is mixed with the delivery solution and applied directly to the cells using an atomizer. After a short incubation period, permeabilization is halted by incubating the cells in a phosphate buffer saline solution that dilutes the ethanol and is non-toxic to the permeabilized cells. Normal culture medium is then added. The procedure lasts less than 5 min. With this method, proteins, mRNA, plasmid DNA and other molecules have been delivered to a variety of cell types, including primary cells, with low toxicity and cargo functionality has been confirmed in proof-of-principle studies. Co-delivery of different cargo types has also been demonstrated. Importantly, delivery occurs by diffusion directly into the cytoplasm in an endocytic-independent manner. Unlike some other vector-free methods, adherent cells are addressed *in situ* without the need for detachment from their substratum. The method has also been adapted to address suspension cells. This delivery method is gentle yet highly reproducible, compatible with high throughput and automated cell-based assays and has the potential to enable a broad range of research, drug discovery and clinical applications.

## Introduction

Delivery of molecules into living cells is highly desirable for a wide range of both research and clinical applications. In a recent comprehensive review of current strategies, Langer and colleagues evaluated the strengths and weakness of these strategies and highlighted features required of “next generation” intracellular delivery systems that include universal application across cell types and delivery materials, compatibility with different target sites within the cells, minimal cell perturbation, and control of dosage [1]. Additional requirements included scalability and reduced cost and complexity of production.

Current methods achieve intracellular delivery under specific conditions, but generally fail to meet most of the goals described above. For example, organic solvents such as dimethyl sulfoxide (DMSO) have been used to deliver cell-impermeant small chemical molecules by permeabilizing the cell membrane [2]. However, such methods are not efficient for larger biological molecules for which vectors or carrier molecules are typically used. Viral- and chemical vector-based methods are widely used to deliver nucleic acid cargoes to cells [3–6]. However, many cell types, particularly primary cells and stem cells, remain difficult to transfect and high toxicity levels are often a problem. Viral vectors for DNA delivery for clinical applications also present many difficulties with regard to safety and production. Furthermore, these methods in general are not well-suited for intracellular delivery of proteins and peptides. Cell-penetrating peptides (CPPs) have been used as vectors to facilitate the uptake of otherwise cell-impermeant peptides and proteins [7]. However, several issues make this a problematic approach. Different CPPs employ varying modes of uptake and the nature of both the cargo and the linker used to conjugate the cargo and CCP can also affect the mode of uptake, efficiency of cellular penetration and internal trafficking [8]. Despite the promise of some of these vector- and carrier-mediated methods, there is a clear need for novel approaches that are closer to meeting the requirements for future applications as outlined in the recent review of intracellular cargo delivery which, in particular, points to membrane-disrupting-based modalities as attractive candidates for universal delivery and large scale production [1].

Membrane-disruption-mediated methods that enable intracellular delivery of various cargo types for clinical applications have potential benefits from several standpoints including safety, regulation and production. Examples include electroporation, magnetofection and intracellular injection methods [6]. Electroporation is the most widely used vector-/carrier-free method but, while it can be efficient for delivery of nucleic acids to some cell types, toxicity can be high, particularly in primary cells. Alternative membrane-disrupting methods are therefore required. One such method was reported recently whereby cells are mechanically deformed when passing through a narrow constriction such that transient membrane disruptions are produced that facilitate passive diffusion of a cargo into the cell [9]. Another method uses a combination of one of the oldest methods to disrupt the cell membrane (exposure to a hypotonic solution to cause cell swelling) and a transduction compound (propane-betaine) to deliver proteins [10].

We have approached the development of a vector-/carrier-free method for intracellular delivery of a broad spectrum of cargos by combining exposure to a permeabilizing solution with modified strategies for both applying the permeabilizing solution to the cells and inducing reversible cell membrane permeabilization. As others have done [11], we started with the following hypotheses: firstly, permeabilization could be induced by treating cells with a solution containing chemicals to modify the cell membrane and secondly, extracellular cargo would subsequently diffuse into the cell as the cells swell due to influx of water. In addition, we hypothesized that cell survival could be enhanced if exposure of the cells to the permeabilizing solution could be carefully controlled in order to allow the permeabilization to be reversed.

The majority of existing chemical permeabilization methods are not aimed at reversible permeabilization. Chemicals typically used in these methods include alcohols, detergents and enzymes. While a small number of studies have reported successful reversible permeabilization using detergents [11, 12], our preliminary results failed to deliver cargoes of interest using these methods and toxicity was a major problem. However, we note that Medepalli and colleagues described the delivery of quantum dots into cultured cells by incubating cells for 5 min at 4°C in a hypotonic physiological buffered solution termed ‘S buffer’ (78 mM sucrose, 30 mM potassium chloride, 30 mM potassium acetate, 12 mM HEPES [11]). Here we describe a novel method for intracellular delivery by means of reversible permeabilization using a low level of ethanol as a permeabilizing agent with a delivery solution modified from S buffer. While ethanol concentrations greater than 30% are known to rapidly permeabilize cells by introducing transient defects in the plasma membrane, concentrations less than 30% cause thinning of the plasma membrane rather than permeabilization [13, 14]. Maintenance of cell viability while achieving efficient influx of cargo into cells is attained by applying the permeabilizing solution in the form of a spray. A subsequent step wherein cells are incubated with a non-permeabilizing phosphate buffered saline (PBS) solution further enhances uptake and viability. With this method, a broad spectrum of molecule types including proteins, mRNA and DNA can be efficiently delivered to adherent and suspension cells in a gentle yet robust and highly reproducible manner. Co-delivery of diverse cargoes is also possible. Importantly, we demonstrate that loading of target cells with cargo occurs in an endocytic-independent manner, unlike other methods including lipofection and hyperosmotic loading [10]. The process is compatible with scaling and automation for high-throughput applications.

## Materials and methods

### Cell culture

A549 human lung cell line (Sigma Aldrich, Cat. No. 86012804) was routinely cultured in Dulbecco's Modified Eagle Medium (DMEM) (Gibco) supplemented with 5% fetal bovine serum and 2 mM L-glutamine (Gibco). Chinese Hamster Ovary (CHO) cell line (American Type Culture Collection (ATCC), CCL-61) was routinely cultured in DMEM (Gibco) supplemented with 10% fetal bovine serum (Fetal Clone II) (Hyclone) and 2 mM L-glutamine (Gibco). Jurkat human leukemic T-cell lymphoblast cell line (Sigma Aldrich, Cat. No. 88042803) was routinely cultured in RPMI 1640 (Gibco) supplemented with 10% (v/v) heat-inactivated fetal bovine serum (Sigma-Aldrich), and 2 mM L-glutamine (Gibco). Human multiple myeloma U266 (U266B1) cell line (ATCC, ATCC^®^TIB-196™) was routinely cultured in RPMI 1640 (Gibco) supplemented with 10% (v/v) heat-inactivated fetal bovine serum (Sigma-Aldrich), and 2 mM L-glutamine (Gibco). Primary fibroblasts (Caltag Medsystems, Cat. No. HDFs.05) were cultured in low glucose DMEM (Sigma Aldrich) supplemented with 10% (v/v) fetal bovine serum. Research involving human mesenchymal stem cells (MSC) was approved by the Institutional Review Boards and Biological Ethics Committee of the National University of Ireland, Maynooth. All human participants provided written informed consent. Human MSC were generated as previously described [15] and conformed to criteria established by the International Society for Cellular Therapy. MSC were cultured in DMEM (Sigma-Aldrich) supplemented with 10% (v/v) fetal bovine serum (Biosera) and 100 U/ml penicillin and 100 μg/ml streptomycin (Sigma-Aldrich) and were used between passages 3 and 10. All cells were maintained at 37°C in a humidified atmosphere of 5% CO_2_. For delivery experiments, cells were seeded at a concentration which yielded 80–95% confluency at 24 hr post-seeding.

### Cargos

Molecules delivered included propidium iodide (Sigma), 4',6-diamidino-2-phenylindole (DAPI) (Molecular Probes), Mitotracker Red CMXRos (Molecular Probes), phalloidin (Molecular Probes), fluorescein isothiocyanate- (FITC-) or Alexa-488-labelled dextrans (Invitrogen), anti-rabbit Alexa-488-labelled secondary antibody (Molecular Probes), bovine serum albumin- (BSA-) FITC (Sigma Aldrich), ovalbumin-FITC (Sigma Aldrich), beta-lactoglobulin (Sigma Aldrich), catalase (Sigma Aldrich), apoferritin (Sigma Aldrich), green fluorescent protein- (GFP) and Gaussia luciferase- (Gluc) encoding mRNA (TriLink Biotechnologies), GFP-encoding plasmid DNA (Clontech) and Gluc-encoding plasmid DNA (New England Bio labs). For in-house FITC labelling of proteins: lyophilized proteins were dissolved in carbonate buffer (0.25 M NaCO_3_; pH 9.3) while proteins already in solution were desalted on a Sephadex G-25 column that had been pre-equilibrated with carbonate buffer. FITC was dissolved in DMSO and diluted to 1 ml with carbonate buffer. The required volume of diluted fluorophore (molar ratio fluorophore to protein, 20:1) was then immediately added drop-wise to the protein solution. The labelling reaction was incubated for 3 hr in the dark at room temperature with constant gentle stirring. Unreacted fluorophore was separated from the FITC-labelled protein conjugate by passage through a column of Sephadex G-25 (0.4 cm x 42 cm; medium grade; Sigma) which had been previously equilibrated with PBS (pH 7.4).

### Delivery solution

The delivery solution (DS) consisted of 32 mM sucrose, 12 mM potassium chloride, 12 mM ammonium acetate, 5 mM HEPES and 25% ethanol in molecular grade water (all from Sigma-Aldrich). Prior to addition of ethanol, the solution was adjusted to pH 7.4 and filter-sterilized. In the case of varied ethanol concentration or cargo volume, the water volume was adjusted accordingly.

### Spray instrument

The atomizer used in the spray instrument was a MAD Nasal™ intranasal mucosal atomization device (Wolfe Tory Medical Inc, Salt Lake City, USA). The atomizer was held on a retort stand and was connected to a 6 bar compressor (Circuit Imprimé Français, Bagneux Cedex, France) via polyurethane tubing (6 mm outside diameter, 4 mm inside diameter; SMC, Tokyo, Japan). The spray was generated using a spray actuator button (SMC, Tokyo, Japan).

### Delivery procedure

For delivery to adherent cells, cells were seeded in 24- or 48-well culture plates (Nunc) at densities that achieved 80–95% confluency at time of delivery. Supernatant was removed from the target well and the culture plate was placed at a distance of 31 mm below the atomizer. The delivery solution, with cargo as appropriate, was pipetted into the delivery port located at the top of the atomizer and was sprayed onto the cells. For 24- or 48-well plates, 20 μl or 10 μl delivery solution respectively was sprayed per well. After 2 min at room temperature, 100 μl 0.5X-PBS (68.4 mM sodium chloride, 1.3 mM potassium chloride, 4.0 mM sodium hydrogen phosphate, 0.7 mM potassium dihydrogenphosphate) was added onto the cells using a micropipette and cells were incubated at room temperature for 30 sec. The PBS solution was removed and fresh culture medium was added. After 3 hr, 100 U/ml penicillin and 100μg/ml streptomycin was added to each well. In the case of double spray, the procedure was repeated after 2 hr incubation from the first spray treatment.

For delivery to suspension cells, 1.0x10^6^ cells were placed into a 0.4 μm polyester membrane insert (Corning). The insert was placed into an in-house vacuum instrument and a vacuum of between -0.5bar and -0.68 bar was applied to remove the culture medium. The insert was then placed into a 12-well plate and positioned under the atomizer. The spray procedure was carried out as described for adherent cells and when fresh culture medium was added, the cells were transferred to a fresh culture plate.

For micropipette delivery controls, the supernatant was removed from the well and cargo in delivery solution was added using a micropipette. After 2 min, delivery solution was removed and fresh culture medium was added. Negative controls for all experiments included untreated cells and cells sprayed with delivery solution only. All experiments were carried out in triplicate.

For delivery experiments involving mRNA and plasmid DNA the commercial transfection reagent Lipofectamine 2000 was used as positive control, as per manufacturer’s instructions. Briefly, 0.5 μg RNA or DNA was combined with 0.5 μl lipid solution in Opti-MEM medium (Gibco) and incubated on cells for 4–6 hr before addition of culture medium.

### Fluorescence and confocal microscopy

Fluorescence analysis was carried out using either the Olympus CKX41 microscope (Optika Vision Pro) and fluorescence module (CoolLED pE-300white) or the Olympus Fluor-View FV1000 confocal laser scanning microscope (Olympus) using the Olympus FluoView software package.

### Flow cytometry

Cells were harvested, centrifuged at 500 x *g* and resuspended in 150 μl PBS (136.8 mM sodium chloride, 2.7 mM potassium chloride, 8.1 mM sodium hydrogen phosphate, 1.5 mM potassium dihydrogenphosphate). Fluorescence was detected using the BD Accuri™ C6 flow cytometer. 10,000 events per sample were acquired. Data analysis was performed using CFlow Plus software (BD Biosciences). The M-line was set to 5% (or 90% confidence interval) on negative control samples and overlaid on analyzed samples to calculate % positive uptake.

### Lactate Dehydrogenase (LDH) assay

The LDH Cytotoxicity Assay Kit (Pierce) was used. At 24 hr post-spray, 50 μl cell supernatant was transferred to a well of a 96-well plate and 50 μl LDH reaction mix was added. The mixture was incubated for 30 min at room temperature in the dark before the addition of 50 μl stop solution. Absorbance was measured at 490 nm and 680 nm. A positive control LDH value for 100% cell death was obtained by repeated freeze-thaw of cells. To calculate % LDH release, the actual LDH value was expressed as a percentage of the total LDH for each sample.

### Electroporation

Electroporation was performed using the Neon™ Transfection System (Thermo Fisher). A549 cells were grown to 70–80% confluency in a T75 tissue culture flask, trypsinized and washed once in PBS. 5 x 10^4^ cells were transferred to a 1.5 ml tube and resuspended in 10 μl Resuspension Buffer R (10 μl Neon Kit) containing 3 μM 10 kDa dextran-Alexa488. Electroporation was carried out under the following conditions; 1200 voltage, 20 width (ms) and 4 pulses. Cells were then seeded into a 24 well culture plate with culture media and analysed by flow cytometry 2 hr after electroporation.

### Immunofluorescence

Cells were cultured overnight on plastic tissue culture coverslips (Sarstedt) and 2 μg ovalbumin-FITC was delivered as described above. Cells were fixed with 3% paraformaldehyde and permeabilized in 0.25% Triton X-100. A rabbit anti-chicken anti-ovalbumin polyclonal antibody (Cat. No. ab181688; Abcam) at 1/1,000 dilution and anti-rabbit Alexa-594 secondary antibody were used to immunolocalize intracellular ovalbumin. Fluorescence was detected using the Olympus CKX41 microscope.

### Gaussia Luciferase assay

The BioLux^®^ Gaussia Luciferase Assay Kit (New England Biolabs) was used. At 24 hr post-spray, 20 μl supernatant was transferred to a well of a 96-well plate and 50 μl GLuc substrate was added. Luminescence was read using a GloMax^®^-Multi Detection System (Promega).

### DNA analysis

DNA quantification was carried out using the Nanodrop (ThermoScientific). DNA samples were run on a 1% electrophoresis gel and analyzed using a Gel Doc™ XRS System and Quantity One^®^ software (Bio-Rad Laboratories).

### Tyramide assay

Cells were grown on plastic tissue culture coverslips and delivery of 8 μg horseradish peroxidase (HRP) was performed as described above. Negative controls consisted of delivery of 8 μg ovalbumin and/or delivery solution only. At 3 hr post-delivery, cells were fixed with 3% paraformaldehyde for 20 min at room temperature and permeabilized using 0.25% Triton X-100 in PBS for 5 min. The Tyramide Signal Amplification (TSA) kit (Molecular probes) was used to detect HRP activity as per manufacturer’s instructions. Cells were rinsed with PBS and 100 μL of tyramide working solution was added to the cells and incubated for 10 min at room temperature. Cells were rinsed with PBS and viewed using the Olympus CKX41 microscope.

### DCFH-DA assay

HRP (8 μg) was delivered to cells as described above. Cells were washed with PBS and incubated overnight in culture medium containing 2 μM dichlorofluorescein diacetate (DCFH-DA). After 24 hr, the solution was diluted in PBS (1:10) and the fluorescence was measured using the POLARstar^®^ Omega spectrofluorometer (BMG LABTECH).

### Cell permeabilization analysis

Propidium iodide (PI) exclusion was used to study cell permeabilization. At different time point post-spray, culture medium was aspirated from wells and 50 μl 0.1 mg/ml PI in PBS was added and incubated for 1 min. The PI solution was then removed and cells were trypsinised, centrifuged and resuspended in PBS as previously described for flow cytometry analysis.

### Inhibition of active uptake pathways

A549 cells were seeded at 2.5x10^4^ cells per well of a 96-well culture plate to achieve a confluency between 80–95% for delivery the following day. An hour before cargoes were delivered, the supernatant was removed and fresh media containing one of the inhibitors was added to the following final concentrations as reported by D’Astolfo and colleagues [10]: Dynasore hydrate, 40 μM; Chloropromazine, 20 μM; Nystatin, 21.6 μM; 5-(N-Ethyl-N-isopropyl)amiloride (EIPA), 100 μM (all from Sigma Aldrich). Cells were then incubated for 1 hr. A vehicle control comprising 2% DMSO was also used and all conditions were carried out in triplicate. After 1 hr, the supernatant was removed and 4 μg enhanced green fluorescent protein (EGFP) mRNA in 10 μl delivery solution was delivered. Flow cytometry analysis was carried out at 24 hr. As a positive control, dynasore hydrate was used to block liposome-mediated delivery, which functions via both clathrin- and caveolar-mediated endocytosis [16]. Lipofectamine 2000 (Invitrogen) was used according to manufacturer’s instructions to deliver EGFP mRNA to A549 cells. An hour before cells were treated with the lipofectamine mixture containing EGFP mRNA, culture medium was replaced with fresh medium containing dynasore hydrate. After 1 hr, supernatant was removed and lipofectamine mixture was added. After 6 hr incubation at 37°C, supernatant was removed and replaced with fresh A549 culture medium. GFP protein expression was evaluated at 24 h by means of flow cytometry as previously described.

### Data analysis

Data are depicted as the mean ± standard deviation (SD).

## Results

### Development of reversible permeabilization delivery solution

In the supplementary information we describe preliminary results using the hypotonic ‘S buffer’ of Medepalli *et al*. which used the detergent saponin as the permeabilizing agent to achieve intracellular delivery of quantum dots [11]. We did not achieve uptake of biomolecules such as siRNA into A549 cells under these conditions and observed high levels of cell damage (S1 Table). These investigations also included tests to develop a reversible permeabilizing protocol using ethanol. A range of ethanol concentrations (0–93%) in several diluents including water, PBS or modified ‘S buffer’ solutions containing various concentrations of sucrose (0–121 mM), potassium chloride (0–46 mM), potassium acetate (0–46 mM), ammonium acetate (0–46 mM) and HEPES (0–19 mM) were examined. Propidium iodide (PI) was used as a cell-impermeant model drug and indicator of cell permeabilization due to its dramatically increased fluorescence upon entering the cell and interacting with nucleotides. However, while PI-positive cells could be indicative of either reversible (viable cells) or irreversible (non-viable) permeabilization, we observed that uptake of larger molecules such as 10 kDa dextran-Alexa488 only occurred when cells remained viable and so were useful indicators of successful reversible permeabilization. Following ranging experiments, we found that the delivery solution composition which gave the best balance between delivery efficiency and cell viability was 75% H_2_O, 25% ethanol, 32 mM sucrose, 12 mM potassium chloride, 12 mM ammonium acetate and 5 mM HEPES and this was used from this point on unless otherwise stated.

### Cargo delivery via direct administration of permeabilizing solutions

**Fig 1:**
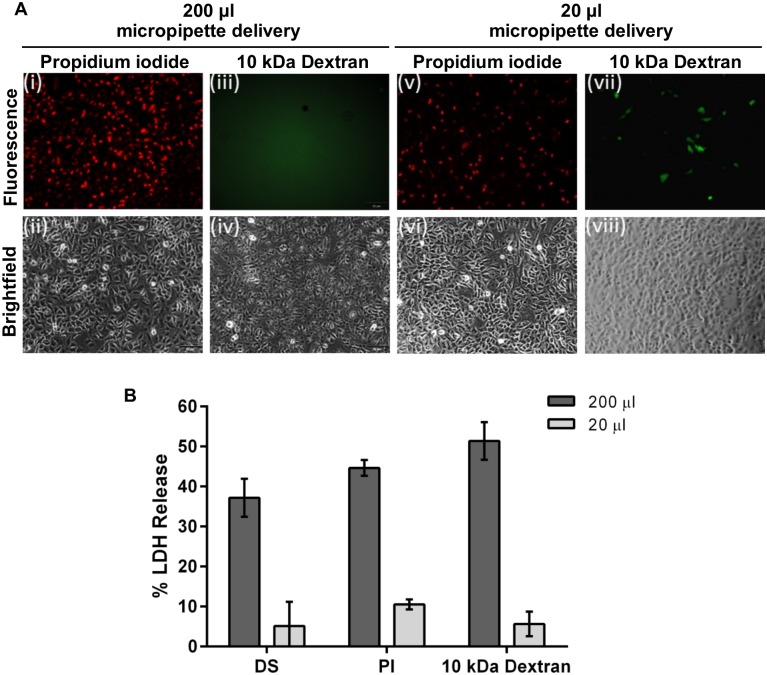
Delivery solution. (A) 150 μM PI or 3 μM 10 kDa Dextran-Alexa488 in 200 μl delivery solution was delivered to A549 cells using a micropipette. Immediately after delivery, uptake of PI was visible throughout the cell population but no uptake of dextran was apparent. With 20 μl delivery solution, PI uptake was apparent where the solution first landed in the well (drop zone) but not in other areas. Low level uptake of 10 kDa Dextran-Alexa488 was also observed in the drop zone. (B) LDH release measured at 24 hr post-delivery indicated that 37.2±4.8%, 44.6±1.9% and 51.4±4.7% cells were damaged when 200 μl delivery solution alone, delivery solution containing PI or delivery solution containing 10 kDa Dextran-Alexa488 respectively was applied. In contrast, LDH release was 5.1±6.0, 10.5±1.3% and 5.6±3.1% respectively for these solutions when a 20 μl volume was applied. All photomicrographs are 10x magnification. *n* = 3, data are depicted as the mean ± standard deviation. (DS = delivery solution only; PI = propidium iodide; LDH = lactate dehydrogenase).

We commenced our studies using the conventional pipette-mediated mode of application of permeabilizing solutions. When a volume of 200 μl delivery solution (per well of a 24-well plate) containing 150 μM PI as cargo was micropipetted directly onto a monolayer of A549 cells, most cells immediately stained positive for PI (Fig 1A(i) and 1A(ii)). However, when a 200 μl volume of larger molecules such as 10 kDa dextran-Alexa488 (3 μM) was applied, delivery was not observed (Fig 1A(iii) and 1A(iv)). LDH release measured at 24 hr post-delivery indicated that approximately 35–50% of cells were damaged when delivery solution alone or containing cargo was applied in a 200 μl volume (Fig 1B). We concluded that the cells were over-permeabilized to the point of death whereby PI could pass into the cell but osmotic gradients could not be established to facilitate influx of the larger molecules.

We surmised that, in order to minimize cell death, the maximum volume of cargo must be delivered to the cell monolayer in the smallest volume and shortest time practicable. The volume of delivery solution was therefore reduced to 20 μl, pipetted onto cells and incubated for 2 min at room temperature (RT). To limit the exposure of cells to the ethanol and thereby reduce toxicity, a subsequent ‘Stop’ step comprising a 30 sec incubation in 0.5X PBS, was included. With this method, PI uptake was apparent but was localized to cells in the ‘drop zone’ under the pipette tip where the delivery solution first landed in the well (Fig 1A(v) and 1A(vi)) and was not evident throughout the rest of the monolayer. When a 20 μl volume of 10 kDa dextran-Alexa488 was applied, low levels of uptake were observed in the drop zone (Fig 1A(vii) and 1A(viii)) but not in the rest of the cell monolayer over which the solution subsequently spread. Importantly, toxicity was reduced to less than 10% with this method (Fig 1B). We surmised that with this procedure, some cells in the drop zone were being reversibly permeabilized but many were over-permeabilized while cells outside the drop zone were under-permeabilized.

### Development of spray method

**Fig 2:**
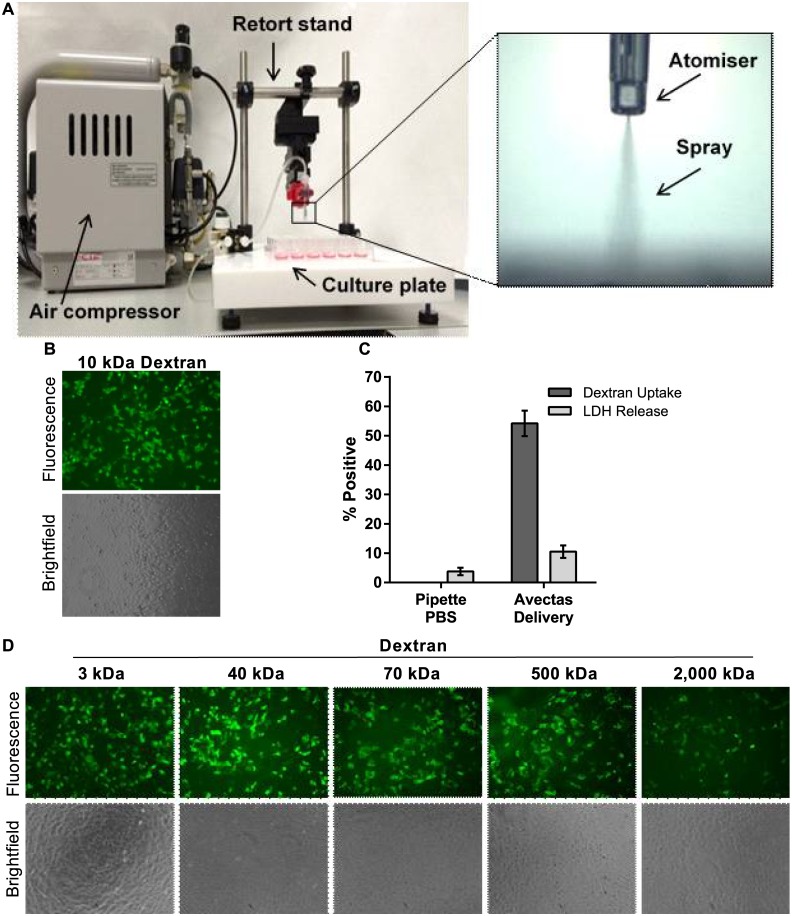
Spray method. (A) The spray instrument comprised of an air compressor that delivered compressed air to an atomizer which was held in position on a retort stand. The culture plate was positioned on a stage below the atomizer. The atomizer could be moved vertically to adjust the distance between the atomizer and the cells. The air pressure levels could also be adjusted. Insert shows the atomizer and spray. (B) 10 kDa dextran-Alexa488 uptake was apparent in A549 cells and uptake was evenly distributed across the cell monolayer. (C) Delivery efficiency levels in A549 cells. Also LDH release was similar to PBS pipette controls. No delivery was observed in controls where 10 kDa dextran-Alexa488 was delivered in PBS using a micropipette. (D) A range of low and high molecular weight dextrans were successfully delivered to A549 cells using the method, 10x magnification. *n* = 3, data are depicted as the mean ± standard deviation. (LDH = lactate dehydrogenase).

We hypothesized that simultaneous administration of the permeabilizing solution to all cells within the monolayer was preferable to ‘dropping on’ small volumes using a micropipette where not all cells could be addressed simultaneously. Furthermore, the volume received by each cell should be titred to permit influx of solution into the cell but be insufficient to bring the cell to the point of cytolysis. We postulated that delivery of the solution in the form of a spray could achieve these outcomes whereby the spray would maximize contact of the cargo with the plasma membrane of the target cells in a very short timeframe and in a uniform manner across the monolayer. To implement this approach, a spray instrument was configured in-house in which an air compressor provided a pressurized air flow to an atomizer which was held in place by means of a retort stand (Fig 2A). According to the manufacturer, the typical droplet size produced by the atomizer is 30–100 microns. An actuator button was used to release the compressed air into the atomizer to produce the spray.

A549 cells were seeded into 48-well plates and 10μl delivery solution containing 3 μM 10 kDa dextran-Alexa488 was loaded into the atomizer. Supernatant was removed from the target well and the delivery solution was sprayed onto the cells. Following a 2 min incubation at RT, 200 μl 0.5X PBS was added and cells were incubated for a further 30 sec. This solution was then removed and 400 μl culture medium was added. This method resulted in successful delivery of 10 kDa dextran into cells with efficiencies of 54.2±4.4% and with toxicity at 10.5±2.1% (Fig 2B and 2C). We confirmed that dextrans could not enter the cells passively by delivering the dextrans in 1X PBS as diluent using a micropipette and no uptake was detected either microscopically or by flow cytometry (Fig 2C). Using this method, FITC-labelled dextrans of increasing sizes up to 2,000 kDa (concentrations ranging from 0.05–3.0 μM for smaller dextrans) were successfully delivered to A549 cells (Fig 2D).

Several parameters were optimized in the course of developing the delivery method. The area of the well surface to which the spray was delivered was optimized by adjusting spray height and pressure per well, and uptake and low toxicity was optimized by adjusting volume and ethanol concentrations (S1 Fig). Interestingly, different modes of cell death were observed during these studies and this contributed to hypothesis formation. For example, in the studies on delivery volume to cells seeded in 48-well plates, volumes of 5 μl appeared to cause cell death by desiccation due to the insufficient volume applied while 20 μl caused cell swelling and detachment and we believe death in this case was due to excessive osmosis. A delivery volume of 10 μl achieved the optimal balance between insufficient and excessive influx of solution. Thus the spray mode of application allowed a greater level of control over the contact of the delivery solution with the cells compared with micropipette delivery. The parameters that produced optimal 10kDa dextran-Alexa488 delivery efficiencies of approximately 45–50% and minimal toxicity levels of less than 10% for cells seeded in 48-well plates were a distance of 31 mm between the atomizer and the cells, a spray pressure of 1.5 bar, an ethanol concentration of 25% and a volume of 10 μl.

### Delivery and viability compared with electroporation

Electroporation is a widely used method for vector-free intracellular delivery. We therefore compared delivery efficiency and cell viability levels using the reversible permeabilization delivery method with electroporation.

**Fig 3:**
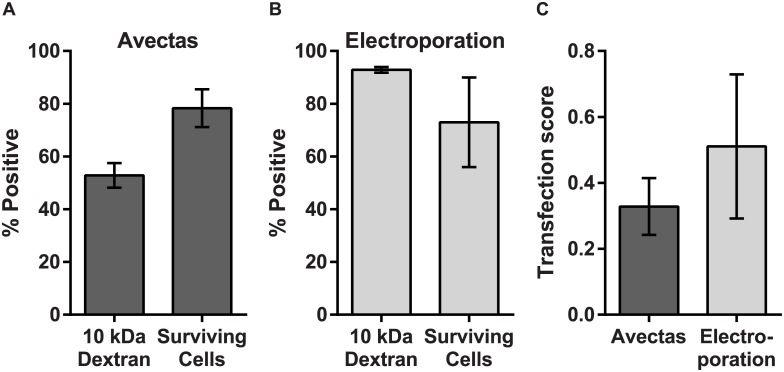
Delivery and viability compared with electroporation. Comparison of delivery efficiency (using 10kDa dextran-Alexa488) and cell viability and survival (using propidium iodide exclusion) for (A) the reversible permeabilization method and (B) electroporation. (C) The transfection score defined as (transfected cells/ total cells)x(viable cells/ total cells) for the two methods). *n* = 3, data are depicted as the mean ± standard deviation.

When 3 μM 10 kDa dextran-Alexa488 was delivered to A549 cells using the reversible permeabilization method, delivery efficiency was 52.8±4.7% compared with 92.9±1.1% for electroporation (Fig 3A and 3B). The percentage of cells that survived the delivery process was analysed by propidium iodide exclusion and quantified using flow cytometric analysis. For the reversible permeabilsation method, cell survival compared with untreated control cells was 78.3±7.2%, compared with 73.0±17.0% for electroporation (Fig 3A and 3B). For most delivery methods, effective delivery must be balanced with maintenance of cell viability. In order to examine this balance, a transfection score ((transfected cells/ total cells)x(viable cells/ total cells)) was used to obtain an aggregate characterisation of cell loss, cell viability and transfection efficiency for the reversible permeabilsation method compared with electroporation. A score of 1.0 would indicate 100% transfection efficiency, 100% cell viability and that no cells were lost during the procedure. The transfection score for the reversible permeabilsation method was 0.33±0.09 and for electroporation was 0.51±0.22 (Fig 3C).

### Delivery of diverse cargos

**Fig 4:**
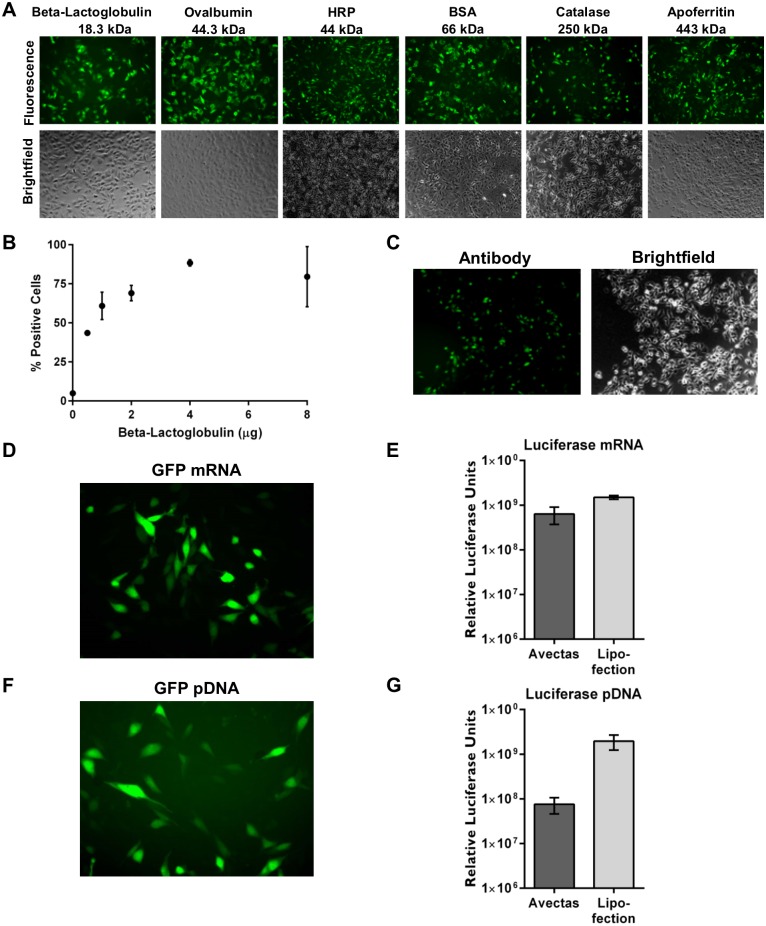
Examples of delivery of diverse cargoes to CHO cells. (A) Proteins of varying sizes were labelled with FITC and 4 μg protein per well was delivered to CHO cells and analyzed by fluorescence microscopy at 2 hr post-delivery, 10x magnification. (B) Increased efficiency of delivery of beta-lactoglobulin was demonstrated with increasing concentration of protein delivered. (C) Full length anti-rabbit-Alexa488 secondary antibody was successfully delivered. (D) GFP mRNA (5 μg) was delivered twice (10 μg/well in total) into cells using the permeabilization method and expression of GFP protein was observed by fluorescence microscopy at 24 hr post-delivery. (E) Luciferase mRNA (5 μg) was delivered twice (10 μg/well total) into cells using the permeabilization method and expression of luciferase was quantified by luminometry at 24 hr post-delivery. For lipofection, luciferase mRNA (5 μg) was delivered per well. (F) pGFP (5 μg) was delivered twice (10 μg/well total) into cells using the permeabilization method and expression of GFP protein was observed by fluorescence microscopy at 24 hr post-delivery. (G) pGLuc (10 μg) was delivered into cells using the permeabilization method and expression of luciferase was quantified by luminometry at 24 hr post-delivery. For lipofection, 0.5 μg pGLuc was delivered per well. *n* = 3, data are depicted as the mean ± standard deviation. (GFP = green fluorescent protein; pGFP = plasmid encoding GFP; pGLuc = plasmid encoding *Guassia* luciferase).

In order to explore the versatility of the method, a variety of biological cargo types and sizes were assessed. A broad range of proteins ranging in size from 18.3 kDa to 443 kDa, were labelled with FITC and delivery into CHO cells was examined by fluorescence microscopy. All proteins were successfully delivered (Fig 4A). The efficiency of uptake of FITC-labelled proteins was determined by flow cytometry and showed that delivery efficiency levels ranged from approximately 30% to 90% for the various proteins (S2A Fig). To further validate cargo entry into cells, ovalbumin-FITC was delivered and intracellular delivery was subsequently confirmed by immunofluorescence using an anti-ovalbumin-Alexa594 antibody (S2B Fig). For a given protein, in this case beta-lactoglobulin, a dose response was evident where increasing efficiency of uptake 43.6±1.5% to 79±19.3% was evident with increasing concentration of protein delivered (Fig 4B). We also examined whether a full length antibody molecule could be delivered by this method and confirmed that an anti-rabbit Alexa488-labelled secondary antibody could be successfully delivered to CHO cells as determined by fluorescence microscopy (Fig 4C).

Reporter mRNAs encoding green fluorescent protein (GFP) and luciferase were delivered into CHO cells. In some cases, two doses of mRNA were delivered to cells with a 2 hr recovery phase between sprays, to increase expression levels. GFP mRNA expression was observed by fluorescence microscopy (Fig 4D). Luciferase mRNA expression was comparable with Lipofectamine 2000 positive controls at 6.4±2.7 x 10^8^ and 1.5±1.4 x 10^8^ relative luciferase units (RLU) respectively (Fig 4E).

Similarly, DNA plasmids encoding GFP (pGFP) and luciferase (pGLuc) were expressed when delivered into CHO cells although levels of pGLuc expression were lower compared with lipofection at 7.6±3 x 10^7^ and 1.9±7.2 x 10^8^ RLU respectively (Fig 4F and 4G). It has been previously reported that jet and ultrasonic nebulizers can cause DNA damage that leads to low DNA transfection efficiencies [17]. We therefore investigated whether our delivery method caused plasmid fragmentation, linearization or single strand nicking using agarose gel electrophoresis. No evidence of plasmid nicking was observed with any treatment (S2C Fig) and the ratio of supercoiled: open circular plasmid in unsprayed versus sprayed samples and in PBS versus delivery solution was unchanged (S2D Fig). These combined data demonstrate the functionality and integrity of nucleic acid cargos following delivery into cells.

### Cell functionality and intracellular targeting

**Fig 5:**
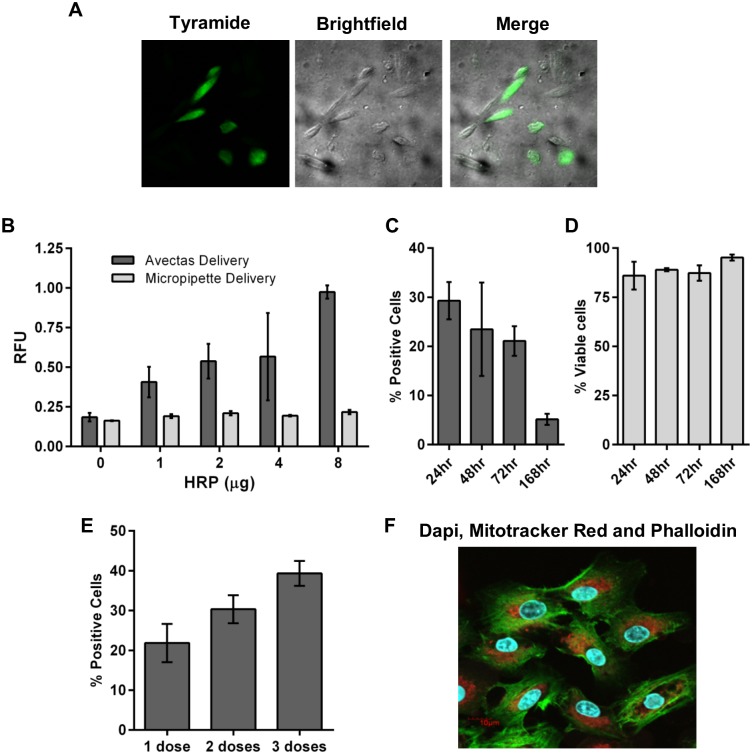
Cell functionality and intracellular targeting. (A) Alexa Fluor^®^ 488-labelled tyramide substrate demonstrated activity and localization of HRP in CHO cells following delivery of HRP. (B) Increasing production of fluorescent DCF product with increasing dose of HRP delivered into CHO cells compared with cells where HRP was delivered by pipette. (C) GFP expression following delivery of GFP mRNA. (D) Cell viability remained above 75% up to 168hr post-delivery. (E) Up to 3 doses of GFP mRNA (4 μg) were delivered. GFP expression was analyzed 24 hr after the final dose. (F) Confocal microscopy image illustrates co-delivery to A549 cells: DAPI (300 nM), Mitotracker Red (50 μM) and Phalloidin-Alexa488 (0.33 μM) correspond to blue nuclei, red mitochondria and green actin filaments, respectively. *n* = 3, data are depicted as the mean ± the standard deviation. (HRP = horseradish peroxidase; DCF = dichlorofluorescein; GFP = green fluorescent protein; DAPI = 4',6-diamidino-2-phenylindole).

The demonstration of mRNA and DNA expression provided proof-of-concept that both cells and cargoes remained functional post-delivery. As further confirmation with other biomolecules, we used two assays to detect horseradish peroxidase (HRP) enzyme activity following delivery of HRP recombinant protein into CHO cells. Firstly, we adapted the Tyramide Signal Amplification (TSA™) assay to demonstrate activity and localization of HRP in CHO cells following delivery (Fig 5A). We also used a DCFH-DA assay to quantify HRP activity. Increasing production of fluorescent dichlorofluorescein (DCF) product was observed with increasing concentration of HRP delivered (Fig 5B). These studies demonstrate that proteins can be delivered directly to cells with functionality retained. Moreover, they demonstrate that cell functionality is retained to facilitate protein expression and function.

We further examined cell functionality and viability by delivering GFP mRNA (4 μg delivered twice with 4 hr between each delivery) to A549 cells and examining GFP expression and cell viability at time points up to 168 hr post-delivery. By flow cytometry, 29.3±3.8% cells expressed GFP at 24 hr post-delivery and by 168 hr, 5.2±1.1% cells were still positive for GFP expression indicating that cells were viable and capable of continued expression of GFP mRNA (Fig 5C). Viability of these cells, as determined by PI exclusion, remained above 75%, demonstrating the continued health of the cells post-delivery (Fig 5D).

The low toxicity of the delivery method is further demonstrated by the ability to administer multiple doses of cargo to cells. When up to 3 separate doses of GFP mRNA were delivered to A549 cells, increased GFP expression was observed (Fig 5E). This is in contrast with techniques such as electroporation where multiple dosing is not possible due to cell death.

The versatility and capability of the method is further illustrated by the ability to deliver combinations of molecules independent of properties and size. For example, DAPI (350 Da), Mitotracker Red (531 Da) and Phalloidin-Alexa488 (1,320 Da) were co-delivered and were observed to localize to the cytoplasm, mitochondria and nucleus respectively (Fig 5F). These data also demonstrate the compatibility of the method with intracellular targeting and the retention of cell functionality post-treatment.

### Delivery to primary cells and suspension cells

**Fig 6:**
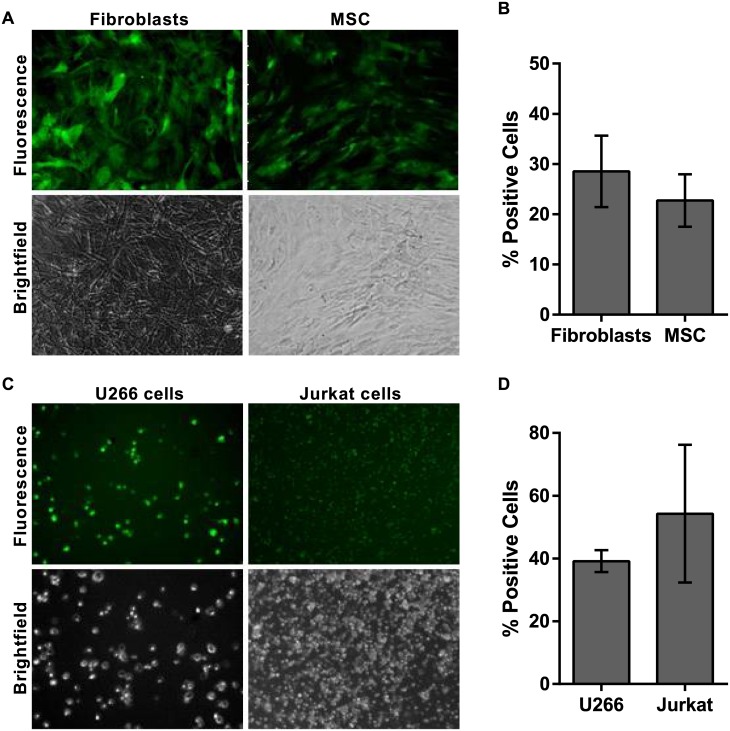
Cell-independent delivery. (A) Delivery of 3 μM 10 kDa dextran-Alexa488 to primary human fibroblasts and primary human MSC. (B) Efficiency of delivery was quantified by flow cytometry at 2 hr post-delivery. (C) Delivery of BSA-FITC to U266 and Jurkat suspension cells. (D) Efficiency of delivery was quantified by flow cytometry at 2 hr post-delivery. All photomicrographs are 10x magnification. *n* = 3, data are depicted as the mean ± the standard deviation. (MSC = mesenchymal stem cells; BSA-FITC = bovine serum albumin-fluorescein isothiocyanate).

The delivery method was successfully deployed across a range of adherent cell types including A549 and CHO lines as described above. The ability to deliver to cells with very low toxicity is important for primary and stem cell populations where large numbers of cells may not be available and minimal manipulation and passaging steps are desirable. Delivery of 10 kDa dextran to primary fibroblasts and primary MSC with low toxicity was successfully achieved (Fig 6A and 6B and S2E Fig).

The method was also successfully adapted to address suspension cells. For these cells, the cell suspension was placed into a porous cell culture plate insert and a brief gentle vacuum of approximately -0.5 to -0.68 bar was applied for 20–45 sec to remove supernatant before delivery to the cells. BSA-FITC was successfully delivered to U266 human multiple myeloma cells and Jurkat cells (Fig 6C and 6D).

### Diffusion of cargo into cells and resealing of plasma membrane

Having demonstrated the ability of this method to deliver a broad range of cargoes to a range of cells types, we then examined the mechanism of cargo uptake into cells and the reversal of the cell permeability. A limitation of other delivery techniques is their dependence on active uptake pathways such as endocytosis which can lead to sequestration of the cargo rendering it unavailable to function in the cell. For example, liposome-mediated delivery involves both clathrin- and caveolar-mediated endocytosis [16] while a contribution of macropinocytosis has also been demonstrated to be involved in protein delivery [10].

**Fig 7:**
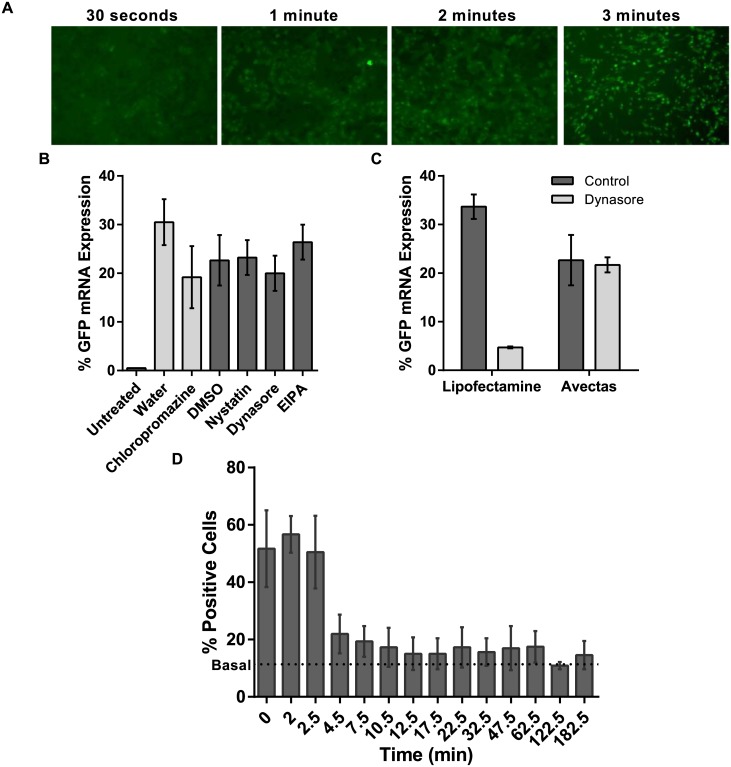
Testing mechanisms of cargo uptake and subsequent membrane resealing. (A) Time course of uptake of 10 kDa dextran-Alexa488 into A549 cells analyzed by fluorescence microscopy consistent with simple diffusion post-delivery (10x mag.). (B) In A549 cells the uptake of EGFP mRNA was not inhibited either by pretreatment with Dynasore or chloropromazine to inhibit clathrin-mediated endocytosis or Nystatin or EIPA to inhibit caveolar-mediated endocytosis and micropinocytosis. (C) Lipofectamine 2000 was used as a positive control for endocytosis-mediated delivery. EGFP expression was reduced in lipofected cells treated with Dynasore. (D) PI uptake was analyzed by flow cytometry and the data indicate that the cells remain permeable to PI for up to 6 min post-treatment but then reseal and prevent uptake thereafter. *n* = 3, data are depicted as the mean ± standard deviation. (EIPA = 5-(N-Ethyl-N-isopropyl)amiloride; EGFP = enhanced green fluorescent protein; PI = propidium iodide; PBS = phosphate buffered saline).

During our experiments, we observed immediate uptake of cargo into cells. Using 10 kDa dextran-FITC as cargo, within 30 sec of applying the delivery solution, before Stop solution was added, cargo was visible within the cells (Fig 7A). The rapid influx of cargo into the cells makes it unlikely that delivery involves endocytosis. Our results showing loading of a wide range of molecular species into a range of cell types suggests that a simple diffusion mechanism through the cell membrane is the most likely mechanism of entry of macromolecules into the cells. To test the contribution of alternate uptake mechanisms such as active pathways and internalization in endocytotic vesicles, A549 cells were pretreated with Dynasore (4 mM) or chloropromazine (20 μM) to inhibit clathrin-mediated endocytosis or Nystatin (21.6 μM) or 5-(N-Ethyl-N-isopropyl)amiloride (EIPA, 100 μM) to inhibit caveolar-mediated endocytosis and micropinocytosis respectively. Expression of EGFP mRNA remained unchanged in the presence of these inhibitors indicating that this method results in direct delivery into the cytoplasm of cells and does not rely on endocytosis (Fig 7B). Furthermore, in addition to following the procedure reported by D’Astolfo *et al*. [10], we included Lipofectamine 2000 as a positive control to confirm Dynasore-mediated inhibition of clathrin-mediated endocytosis (Fig 7C).

We noted that our delivery method was very gentle on cells with little if any cell death or damage evident. We hypothesized that the method allows the permeabilized plasma membrane to reseal rapidly, hence retaining high levels of cell viability. To examine the rate of recovery of the cell membrane after permeabilization, delivery solution was applied to A549 cells in the absence of cargo. At subsequent time points (0 to 182.5 min), this delivery solution was removed and 50 μl PBS containing propidium iodide (150 μM) was added. After 2 min incubation, the PI solution was removed and the cells were harvested. PI uptake was analysed by flow cytometry. For basal levels of PI uptake, untreated cells received 50 μl PI in PBS. The results demonstrate that the cells remain permeable to PI for several minutes but reseal over a period of 6 min post treatment (Fig 7D). After 6 minutes there is no further uptake. Thus not only do the cells load within 2 minutes of exposure to the delivery solution, but the membrane has effectively recovered its integrity within 6 minutes of beginning the procedure.

### Mechanism of action

**Fig 8:**
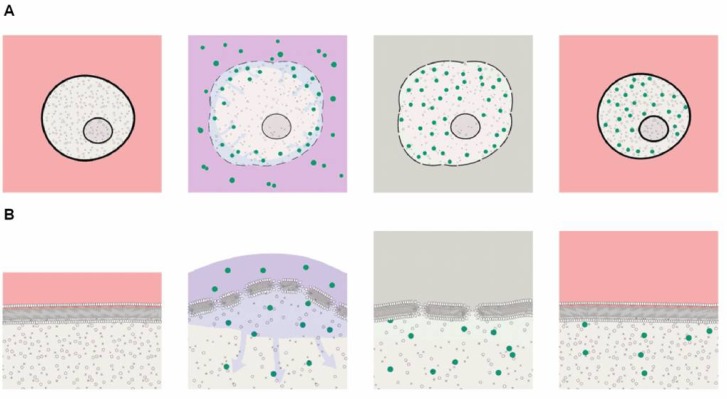
Mechanism of action. (A) Graphic representation of mechanism of action. Cells are initially in culture medium (pink). The cartoon illustrates a sequence lasting about 6 minutes. Ethanol the perturbs cell membrane and makes it susceptible to transient permeablization. Cell begins to swell as extracellular water moves into the cell due to the oncotic effects of the large molecules in the cytoplasm. Cargos now move across the membrane: For smaller molecules, the predominant mechanism would be diffusion. For larger molecules the osmotically-driven water influx (a process known as ‘solvent drag’) augments diffusion by carrying cargo into the cells and concentrating cargo at the cell membrane. Solvent drag may be particularly important for even larger molecules, where an additional tendency for molecules to be carried toward the membrane is a consequence of the spray mode of applying pressure-dependent mechanical force to the cells. We propose that the velocity of the spray droplets leads to a concentration of the larger molecules close to the cell membrane where they enter the cell by diffusion. The next critical step is resealing the membrane and restoration of cell viability. The standard histology fixation protocols use higher ethanol concentrations (>30%) and longer incubation times which result in loss of cell viability due to irreversible membrane permeabilization. Diluting the ethanol more than 50-fold with a PBS solution which is non-toxic to permeabilized cells enables the membrane to reseal (grey). After the cells are returned to culture medium water leaves the cell by the cell’s own regulatory processes as normal electrolyte osmotic gradients are restored. (B) A higher magnification view of the processes at the cell membrane. Exposure to ethanol (25%) thins the membrane so that the tension caused by cell swelling induces reversible permeabilization sufficient to allow entry of cargos as large as proteins and DNA plasmids. Subsequent washout of ethanol restores membrane thickness, reseals the cell membrane and enables recovery in culture medium.

A key feature of this new delivery method is the reversible permeabilization of the cell membranes in the presence of ethanol (Fig 8A). To control the reversibility of the permeabilization, it is necessary to terminate the action of ethanol after exposure of the cells to ethanol at a concentration of 25%. After permeabilization, we achieved maximal cell viability by adding an excess of an ethanol-free solution. The Stop solution step, comprising 50 μl of 0.5X PBS, results in close to a 1 in 50 volume dilution of ethanol and other components of the delivery solution. Our observations also indicated that dilution alone was not the only mechanism contributing to recovery because the use of culture medium as Stop solution compromised viability (data not shown). We suggest that, because the cells are permeabilized at the point the stop solution is applied, components of the Stop solution can enter the cells and may compromise the intracellular electrolyte composition in ways that are toxic to the cells. Thus, the composition of the Stop solution is an important factor in the control of the process.

## Discussion

We describe a novel vector- and carrier-free reversible permeabilization method for achieving intracellular delivery of a broad spectrum of molecule types. While many delivery methods are restricted to certain classes of cargo and are limited by parameters such as size or charge, we have demonstrated that delivery using our method is independent of the molecule type being delivered. Our data demonstrate that the method is not damaging to cells or cargos and functionality of both is retained. Other groups have described reversible permeabilization methods for intracellular delivery [11, 12, 18] but these have not been widely adopted, particularly for biological cargos, and we found toxicity to be a significant problem when we tested these methods. By closely observing the type of damage caused to cells by various permeabilizing solutions and at different stages of the process, we were able to devise strategies to maintain cell viability. We observed that the use of ethanol as a permeabilizing agent at a concentration of 25% gives best results. This is consistent with observations in model membrane systems that ethanol, at concentrations below a threshold of 30.5% v/v (12mol%), partitions into the membrane bilayer and results in expansion of membrane area and reduced membrane thickness, but does not cause breakdown of the bilayer structure and the admixing of membrane components from the two sides of the membrane that leads to irreversible changes. It is particularly notable that the changes in bilayer structure at ethanol concentrations < 10mole% appear to be reversible [13]. It is noted that ethanol, even at higher concentrations, does not form “pores “within the membrane. Thus, the transient membrane disruptions that form to allow the observed uptake of macromolecules under the conditions where small droplets of the delivery solution spread on the cell surface to enable cell loading result, at least in part, from the additional membrane tension caused by the cell swelling.

The lack of evidence for pinocytosis, plus the failure of nystatin, and the dynamin inhibitor dynasore, to modify uptake by caveolae- or clathrin-dependent mechanisms suggests that the simplest mechanism of action for cellular loading is passive diffusion of substances into the cells through reversible membrane disruptions formed during the swelling of the cells exposed to ethanol. With respect to cell swelling, water influx can result not only from osmotic pressure due to differences in the concentration of small solutes and electrolytes but also from the colloid osmotic pressure due to constituent intracellular macromolecules. While osmotic flow due to small solutes would be self-limiting, as it would contribute only while the cell membrane was intact and small solute concentration differences sustained, swelling due to oncotic forces would continue even when the cell membrane was disrupted. We note there is extensive literature over many decades reporting the loading of a variety of agents into red cell ghosts after exposure to only hypotonic solution for loading (see Bourgeaux *et al*. 2016 for current review) [19]. The method described here differs from these approaches because the cells involved are much harder to transfect and are not just passive carriers but retain cellular functions critical for specific clinical and research applications. For macromolecules, further loading may be enhanced by the accumulation of the molecules at the cell surface as they are carried towards the membrane coupled to water flows (solvent drag) caused either by the initial osmotic flow or by the velocity imparted to the spray droplets directed towards the cell surface.

Regarding the role of ammonium acetate in the delivery solution, we hypothesise that ammonia (in equilibrium with ammonium ions: NH_4_ → NH_3_ + H^+^) could enter the cells during the initial exposure to delivery solution tending to increase intracellular pH, although this would be buffered by subsequent diffusion of both NH4 and acetate ions through transient membrane disruptions. The subsequent replacement of the delivery solution with the ammonium free Stop solution (PBS) would favour intracellular acidification as the gradient for NH_3_ in equilibrium with NH_4_ in the cell was reversed and protons accumulated in the cell [20]. Such changes in intracellular pH have been associated with regulation of the actin cytoskeleton [21]. In particular, we noticed that the presence of ammonium acetate was associated with occasional blebbing of the membrane. Whereas blebbing is often associated with membrane damage, there is growing evidence that blebbing can relieve excess membrane tension and contribute to cell recovery [22]. Thus, we speculate that ammonium acetate contributes to cell viability by direct or indirect actions to limit cell membrane perturbations during reversible permeabilization.

The ability to rapidly deliver cargos into cells in situ with a minimal number of steps is highly advantageous for a wide range of applications. When evaluated against the criteria summarized in the Introduction, the strategy described herein appears uniquely promising. With respect to universal application to cargo materials, every type of molecule tested to date has been successfully delivered with this method. While efficiency levels seem to reduce somewhat with larger-sized molecules, nonetheless, large molecules such as plasmid DNA have been successfully delivered and shown to be functional. It is likely that adjustments to certain parameters will lead to increased efficiencies for these larger molecules. Significantly, the activity of proteins, mRNA, plasmid DNA and labeling molecules has been demonstrated following the delivery regime, indicating that both the cargos and the cells remain functional. We have also demonstrated co-delivery of molecules and theoretically, limitless combinations of molecules can be delivered simultaneously.

With respect to the variety of cell types we have demonstrated effective delivery to adherent cell including A549 and CHO lines, as well as to primary fibroblasts and primary MSC. The protocol was also successfully adapted to address suspensions of U266 human multiple myeloma cells and Jurkat cells after they were gently accumulated onto a porous cell culture plate insert and a brief gentle vacuum applied to remove supernatant.

A key feature of the method is the limited number steps involved and the fact that cells are loaded within 5 minutes and reseal 6 minutes after stopping the exposure to the delivery solution containing ethanol. The strategy avoids steps known to decrease cell viability. For example, electroporation, a widely used vector-free delivery method, requires detachment and this introduces additional steps into a process and can provide opportunity for contamination. Additionally, the electric field involved in the electroporation process means that delivery is affected by the charge on the cargo and delivery of proteins is generally inefficient with this method [23, 24]. Furthermore, the electric field can damage both the target cell and the cargo [25, 26]. The data here indicate that our permeabilization delivery method is gentle on cells and on cargo and causes only low levels of cell toxicity.

There is a particular unmet need for effective intracellular delivery methods for peptides, proteins and antibodies in a vector-free, endocytic-independent manner for both research and clinical purposes. The method described here has potential to enable *ex vivo* delivery of these molecules to cell for therapeutic applications. The method requires no specialized equipment or expertise and throughput is similar to that of electroporation. We have also demonstrated co-delivery of molecules and theoretically, limitless combinations of molecules can be delivered simultaneously. This has potential to enable co-delivery of therapeutic molecules for multiple targets in complex diseases under conditions that allow control of the delivery dose as required under research and clinical conditions. Finally, we note that both the delivery procedure and the handling of the cellular material are compatible with scaling and automation for both research and therapeutic applications. In particular, the delivery of therapeutic agents within a time period of 5 minutes is advantageous for use with delicate primary and stem cells and for integration into larger processes.

## Supporting information

S1 Table Assessment of delivery of biomolecules (siRNA-FITC) to A549 cells using method reported by Medepalli *et al*. (2013). (DOCX)

S1 Fig Optimal parameters. Key parameters were varied for the delivery of 3 μM 10-kDa dextran-Alexa488 to A549 cells seeded in 48-well plates. The effect on delivery efficiency and toxicity was determined by flow cytometry at 2 hr post-delivery and LDH release at 24 hr post-delivery, respectively. (A) A concentration of 25% ethanol was optimal with delivery efficiency at 48.9±4.3% and toxicity at 0±0%. (Bb) A volume of 10 μl was optimal with delivery efficiency at 45.2±3.0% and toxicity at 0.5±0.4%. (C) A distance of 31 mm was optimal with delivery efficiency at 45.2%±3.0% and toxicity at 5.1±4.4%. (D) A pressure of 1.5 bar was optimal with delivery efficiency at 45.2±3.0% and toxicity at 3.2±4.6%. *n* = 3, data are depicted as the mean ± standard deviation.
(TIF)

S2 Fig Diverse cargoes. (A) Efficiency of delivery of proteins was analyzed by flow cytometry at 2 hr post-delivery. (B) Immunofluorescence using an anti-ovalbumin antibody confirmed that ovalbumin protein was present in cells following delivery of ovalbumin-FITC (Ova-FITC). (C) Unsprayed (‘no spray’) plasmid DNA was diluted in PBS or delivery solution and the presence of open circular (oc), linearized (lin) and supercoiled (sc) was visualized by electrophoresis on an agarose gel and compared with three separate plasmid DNA samples post-spray. No increase in open circular or linearized plasmids was observed in sprayed samples compared with unsprayed DNA. (D) Densitometry analysis of the agarose gels confirmed that neither the delivery solution nor the spray process adversely affected plasmid DNA integrity compared with control unsprayed ‘No spray’ plasmid. (E) LDH release in primary fibroblasts and MSC was less than 15%. All photomicrographs are 10x magnification. (DS = delivery solution). *n* = 3, data are depicted as the mean ± standard deviation.
(TIF)

## Abbreviations

ATCC: American type culture collection
BLG: beta-lactoglobulin
BSA: bovine serum albumin
CHO: Chinese hamster ovary cell line
CPP: cell-penetrating peptide
DAPI: 4',6-diamidino-2-phenylindole
DCF: dichlorofluorescein
DCFH-DA: dichlorofluorescein diacetate
DMEM: Dulbecco's Modified Eagle Medium
DMSO: dimethyl sulfoxide
DS: delivery solution only
EIPA: 5-(N-Ethyl-N-isopropyl)amiloride
FITC: fluorescein isothiocyanate
GFP: green fluorescent protein
GLuc: *Gaussia* luciferase
HEPES: 4-(2-hydroxyethyl)-1-piperazineethanesulfonic acid
HRP: horseradish peroxidase
KCl: potassium chloride
LDH: lactate dehydrogenase
MSC: mesenchymal stem cell
OVA: ovalbumin
PBS: phosphate buffered saline
PI: propidium iodide
RLU: relative luciferase units
RT: room temperature
TSA: Tyramide Signal Amplification
