# Supplementary material for: Vector-free intracellular delivery by reversible permeabilization
Source: PLoS One. 2017 Mar 30;12(3):e0174779. doi: 10.1371/journal.pone.0174779 (PMC5373627; doi:10.1371/journal.pone.0174779)
Supplement: S1 Table — (DOCX) [file pone.0174779.s001.docx]

**S1 Table: Assessment of delivery of biomolecules (siRNA-FITC) to A549 cells using method reported by Medepalli et al. (2013)**

| **Saponin conc.** | **Incubation Time** | **Estimated**  **% PI uptake** | **Estimated**  **% siRNA-FITC uptake** | **Cell morphology** |
| --- | --- | --- | --- | --- |
| 50 µg/ml | 2 min | 100 % | Not tested | Cell damage |
| 50 µg/ml | 5 min | 100 % | Not tested | Cell damage |
| 50 µg/ml | 10 min | 100 % | Not tested | Cell damage |
| 25 µg/ml | 2 min | 100 % | 0% | Intact monolayer |
| 25 µg/ml | 5 min | 100 % | 0% | Intact monolayer |
| 25 µg/ml | 10 min | 100 % | 0% | Intact monolayer |
| 10 µg/ml | 2 min | 10 % | 0% | Cells appear healthy |
| 10 µg/ml | 5 min | 80-90 % | 0% | Cells appear healthy |
| 10 µg/ml | 10 min | 100 % | 0% | Intact monolayer |

Medepalli et al. 2013 reported that 50 µg/ml Saponin in a 3:2 S Buffer: diH_2_0 for 5 min at 4°C was optimal to induce reversible permeabilization and deliver quantum dots to cells.

For our studies, A549 cells were seeded in 24-well tissue culture plates and were 100% confluent the following day when treated. Delivery solution was 3:2 S Buffer: diH_2_0 (60% Sbuffer, 40% diH_2_0) containing 10 µg/ml, 25 µg/ml or 50 µg/ml saponin. Delivery solution containing either 0.15 M propidium iodide (PI) or 3.3 µM FITC-labelled siRNA (siRNA-FITC) was incubated with cells for 2 min, 5 min or 10 min at 4°C. In brief, supernatant was removed from cells, Saponin-containing solution was added and cells were incubated at 4°C for indicated durations. Cells were then rinsed twice with ‘S Buffer’ and fresh medium was added.

Cell morphology and delivery of cargo was analysed by brightfield and fluorescence microscopy respectively the following observations were made:

At 50 ug/ml Saponin, extreme cell damage was noted. Following rinse steps, approximately 40 % of cells had detached from the well plate. Cells which remained were 100 % brightly stained with PI indicating cell death. This effect was seen at 2 min and enhanced with longer incubation times.

At 25 ug/ml Saponin, cells were visibly permeabilized (flattened appearance via brightfield microscopy) but the monolayer remained intact suggesting less damage compared to that induced by 50ug/ml saponin. 100% cells were stained with PI but no siRNA-FITC uptake was seen. It was hypothesised that cells were over-permeabilized and no osmotic gradient could be established to allow siRNA molecules diffuse into the cells.

At 10 ug/ml Saponin with a 2 min incubation, cells looked less damaged with low levels of PI uptake. At 5 min, approximately 80-90 % cells were stained with PI but no siRNA-FITC uptake was seen at 5 min or 10 min.

We concluded that biomolecules such as siRNA could not be delivered to live cells with this method.
