# Supplementary figures and images for: Vector-free intracellular delivery by reversible permeabilization
Source: PLoS One. 2017 Mar 30;12(3):e0174779. doi: 10.1371/journal.pone.0174779 (PMC5373627; doi:10.1371/journal.pone.0174779)

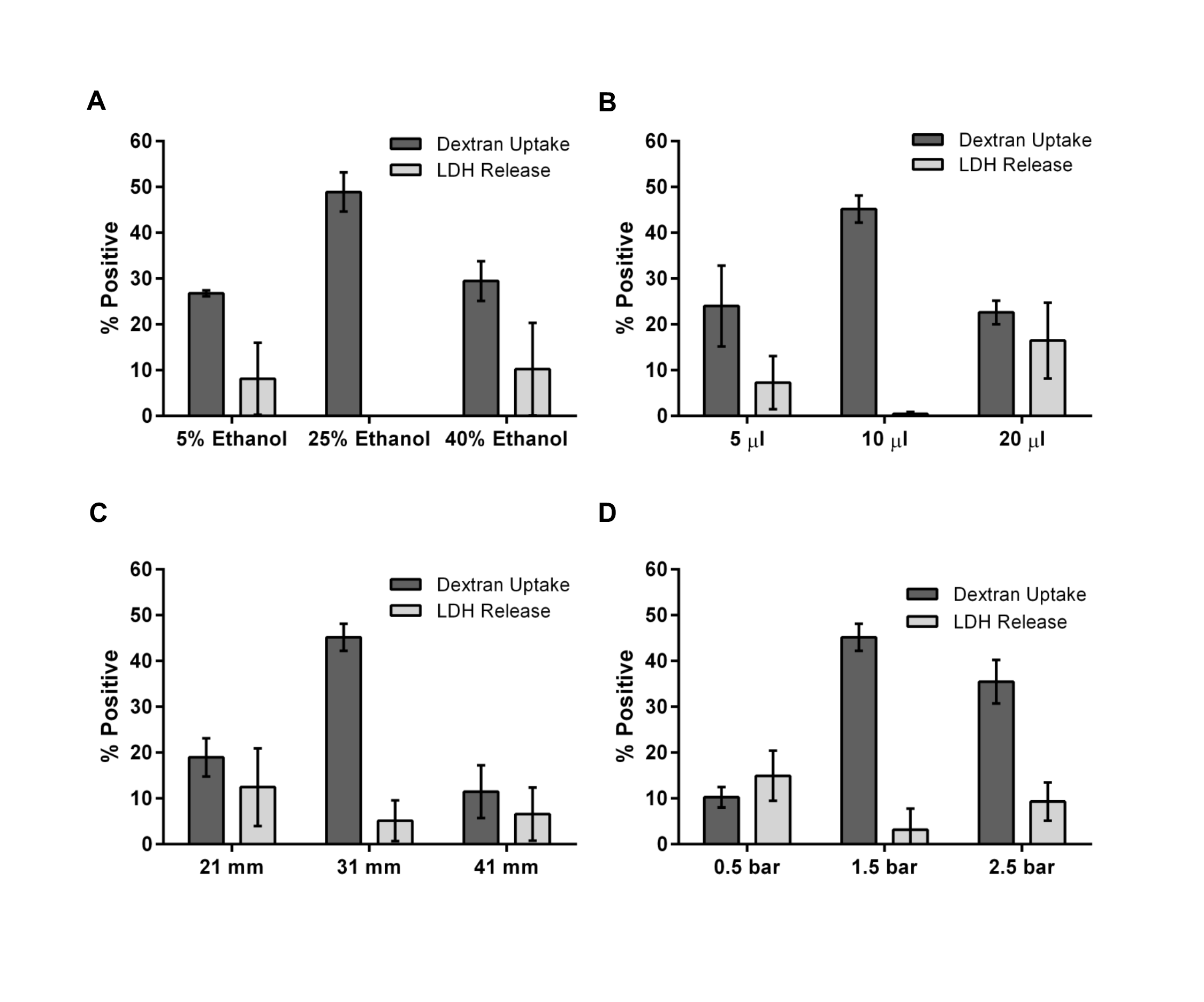

Supplement: S1 Fig — Key parameters were varied for the delivery of 3 μM 10-kDa dextran-Alexa488 to A549 cells seeded in 48-well plates. The effect on delivery efficiency and toxicity was determined by flow cytometry at 2 hr post-delivery and LDH release at 24 hr post-delivery, respectively. (A) A concentration of 25% ethanol was optimal with delivery efficiency at 48.9±4.3% and toxicity at 0±0%. (Bb) A volume of 10 μl was optimal with delivery efficiency at 45.2±3.0% and toxicity at 0.5±0.4%. (C) A distance of 31 mm was optimal with delivery efficiency at 45.2%±3.0% and toxicity at 5.1±4.4%. (D) A pressure of 1.5 bar was optimal with delivery efficiency at 45.2±3.0% and toxicity at 3.2±4.6%. n = 3, data are depicted as the mean ± standard deviation. (TIF) [file pone.0174779.s002.tif]

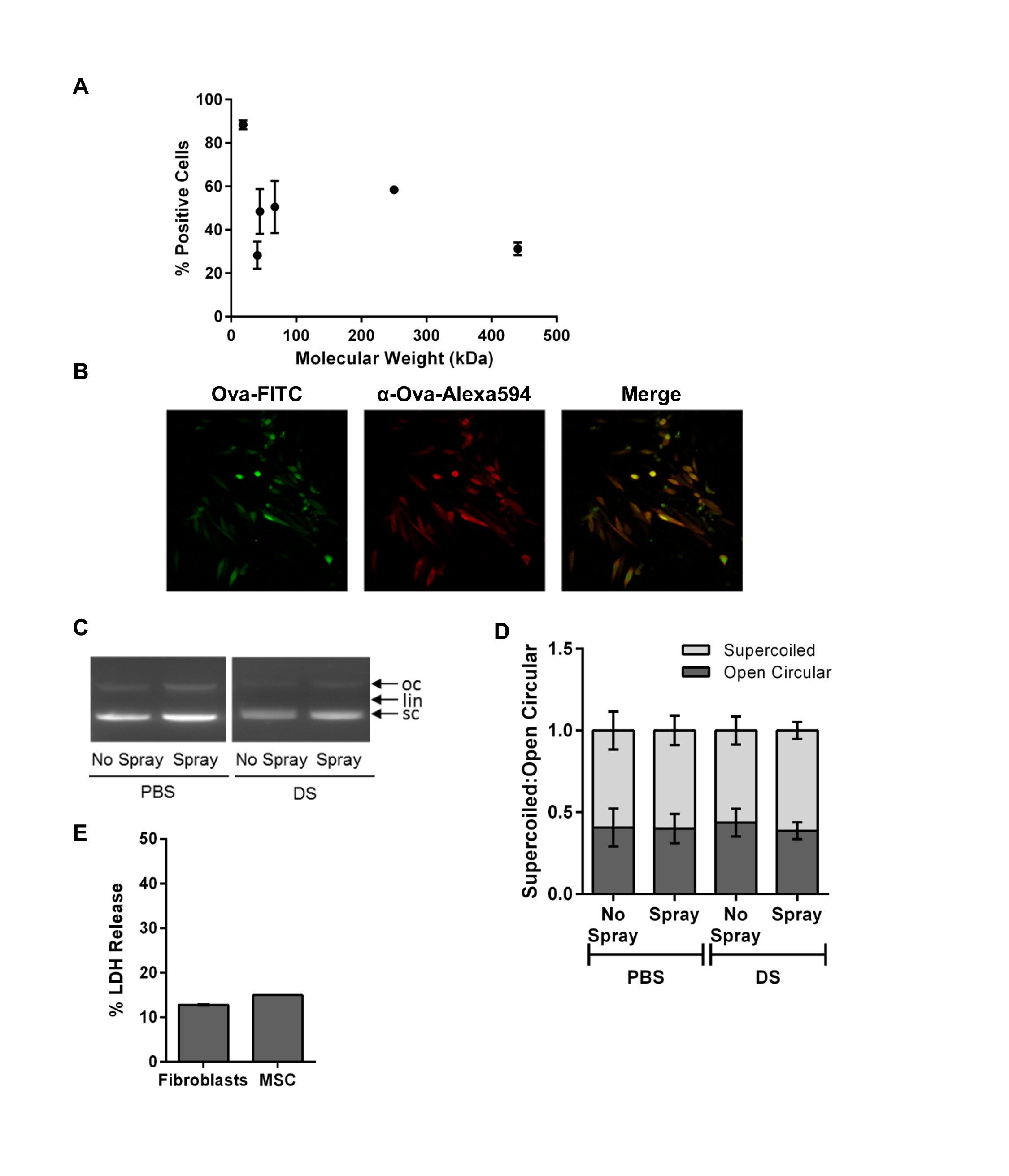

Supplement: S2 Fig — (A) Efficiency of delivery of proteins was analyzed by flow cytometry at 2 hr post-delivery. (B) Immunofluorescence using an anti-ovalbumin antibody confirmed that ovalbumin protein was present in cells following delivery of ovalbumin-FITC (Ova-FITC). (C) Unsprayed (‘no spray’) plasmid DNA was diluted in PBS or delivery solution and the presence of open circular (oc), linearized (lin) and supercoiled (sc) was visualized by electrophoresis on an agarose gel and compared with three separate plasmid DNA samples post-spray. No increase in open circular or linearized plasmids was observed in sprayed samples compared with unsprayed DNA. (D) Densitometry analysis of the agarose gels confirmed that neither the delivery solution nor the spray process adversely affected plasmid DNA integrity compared with control unsprayed ‘No spray’ plasmid. (E) LDH release in primary fibroblasts and MSC was less than 15%. All photomicrographs are 10x magnification. (DS = delivery solution). n = 3, data are depicted as the mean ± standard deviation. (TIF) [file pone.0174779.s003.tif]
